# Statistical inference for ordinal predictors in generalized additive models with application to Bronchopulmonary Dysplasia

**DOI:** 10.1186/s13104-022-05995-4

**Published:** 2022-03-22

**Authors:** Jan Gertheiss, Fabian Scheipl, Tina Lauer, Harald Ehrhardt

**Affiliations:** 1 School of Economics and Social Sciences, Helmut Schmidt University/University of the Federal Armed Forces, Hamburg, Germany; 2 Department of Statistics, Ludwig Maximilians University, Munich, Germany; 3 Department of General Pediatrics and Neonatology, Justus Liebig University, Giessen, Germany; 4 German Center for Lung Research (DZL), Universities of Giessen and Marburg Lung Center (UGMLC), Giessen, Germany

**Keywords:** Chronic lung disease, Logit model, Ordinal data, Regularization, Smoothing penalty

## Abstract

**Objective:**

Discrete but ordered covariates are quite common in applied statistics, and some regularized fitting procedures have been proposed for proper handling of ordinal predictors in statistical models. Motivated by a study from neonatal medicine on Bronchopulmonary Dysplasia (BPD), we show how quadratic penalties on adjacent dummy coefficients of ordinal factors proposed in the literature can be incorporated in the framework of generalized additive models, making tools for statistical inference developed there available for ordinal predictors as well.

**Results:**

The approach presented allows to exploit the scale level of ordinally scaled factors in a sound statistical framework. Furthermore, several ordinal factors can be considered jointly without the need to collapse levels even if the number of observations per level is small. By doing so, results obtained earlier on the BPD data analyzed could be confirmed.

## Introduction

Bronchopulmonary Dysplasia (BPD) is a chronic lung disease often found in preterm infants with lungs not fully developed. Disturbance of lung development and severity of BPD is caused by various peri- and postnatal factors including prematurity itself, as well as pre- and postnatal infections [1]. BPD is measured on ordinal scale with grades 0, 1, 2, 3, but often dichotomized as 0: ‘no/mild BPD’ and 1: ‘moderate/severe BPD’. One goal of the study reported here is to investigate whether the time after birth some specific bacteria were found for the first time in the children’s upper airways has an effect on BPD. Initially, $n = 102$ preterm infants with a birth weight < 1000 g and gestational age $\le$ 32 $+$ 0 weeks were analyzed within a retrospective cohort study at the tertiary perinatal center of Justus-Liebig-University Giessen (Germany) between January 2014 and June 2017. Two infants, however, had to be excluded from further analyses at some point due to missing information on some bacterial colonization. Earlier analyses already showed that the later bacteria were found, the lower the risk of developing BPD [2]. However, it is not fully understood yet which specific bacteria have an effect, and in which way. Therefore we will draw special attention to the time period/week (after birth) three types of bacteria—gram negative/positive and pathogenic—were found for the first time in the upper airway of the respective child. Although ‘time’ is supposed to be a continuous variable, information is only available in a discretized way here, because samples were only obtained once a week. Furthermore, the last category ‘week > 6’ is open/censored. If an observation is falling in the last category, we only know that until week six the respective germ had not been found yet. So, the corresponding covariate may only be considered as categorical but ordinal.

Besides the information on bacteria, some additional risk factors need to be taken into account, such as the weight and sex of the child, the number of days antibiotics and steroids were given, or information on multiples. For doing so, a logit model with categorical/ordinal predictor ‘bacteria/week’ and additional, potentially confounding covariates may be fit. Typically, a categorical predictor is included as dummy-coded factor, ignoring, however, the information on the categories’ ordering (if so). In the case presented, additional problems are caused by the fact that some categories/levels only have a few observations, and sometimes all those observations are falling in the same response category. Consequently, some coefficients in a logit model fit via usual maximum likelihood tend towards $\pm \infty$.

For preventing numerical problems like inflating regression coefficients, penalization can be a viable solution [3]. Furthermore, a penalty term can be used for exploiting/respecting a covariate’s ordinal scale level. Following [4–7], for instance, a difference/smoothing penalty might be put on adjacent dummy coefficients of the ordinal factor when fitting the model. An approach that has already been applied successfully in medical research; see, e.g., [8, 9]. In the BPD application, however, the question naturally arises how to test for significance of the ordinal predictor in the penalized setting. In a linear model with normal errors, this can be done using a (restricted) likelihood ratio test [10–13], after rewriting the ordinal penalty as a mixed model [14–16]. However, the corresponding test is not available for generalized linear models, such as the logit model considered here. In this note, we will illustrate how technology developed for generalized additive models [17, 18] can be used to fit generalized linear and additive models with ordinal smoothing penalty, and conduct further statistical inference.

## Main text

### Methods

Given a response *y* with distribution from a simple exponential family, and a set of covariates $x_1,\ldots ,x_p$, a generalized additive model [17] has the form:

1 $$\begin{aligned} \eta = \alpha + f_1(x_1) + \ldots + f_p(x_p), \;\; \mu = h(\eta ), \end{aligned}$$

where $\mu$ is the (conditional) mean of *y* given the covariates, *h* is a (known) response function, and $\eta$ is comparable to the linear predictor in generalized linear models [19, 20]. The difference to a generalized linear model is that non-linear functions $f_j$, $j=1,\ldots ,p$, are allowed in $\eta$, but still the structure of $\eta$ is additive. Of course, if $f_j$ are restricted to be linear, a generalized linear model is obtained as a special case. In a (generalized) additive model, however, it is usually only assumed that functions $f_j$ are reasonably smooth; and one way to fit such models, as for instance implemented in the popular R package mgcv [18, 21], is to specify a set of basis functions for each predictor and to employ an appropriate, quadratic smoothing penalty on the corresponding basis coefficients. That means, we assume that

2 $$\begin{aligned} f_j(x) = \sum _{r=1}^{q_j} \beta _{jr} B_{jr}(x), \end{aligned}$$

with $B_{j1}(x),\ldots ,B_{jq_j}(x)$ being a reasonably rich set of basis functions chosen for function $f_j$, and $\beta _{j1},\ldots ,\beta _{jq_j}$ are the corresponding basis coefficients. When fitting those basis coefficients to the data, a penalty term $J_j(\beta _j)$ is typically added for each covariate $x_j$, penalizing wiggly basis coefficients and thus wiggly functions $f_j$. The strength of the penalty and hence the amount of smoothing is controlled through a tuning parameter, often denoted by $\lambda _j$.

Now suppose you have a categorical predictor $x_j$ with levels $1,\ldots ,k_j$. Then, there is a somewhat natural basis: the basis of (dummy) functions ($l=1,\ldots ,k_j$)

3 $$\begin{aligned} B_{jl}(x_j) = \left\{ \begin{array}{ll} 1 \quad \text{ if } x_j = l,\\ 0 \quad \text{ otherwise}. \end{array} \right. \end{aligned}$$

Since we know that $x_j$ can only take values $1,\ldots ,k_j$, we do not need to think about the type and number of basis functions, placing of knots, etc., as we usually do with continuous covariates. If now a (quadratic) first-order difference penalty

4 $$\begin{aligned} J_j(\beta _j) = \sum _{l=2}^{k_j} (\beta _{jl} - \beta _{j,l-1})^2, \end{aligned}$$

is put on the basis/dummy coefficients $\beta _j = (\beta _{j1},\ldots ,\beta _{jk_j})^\top$, this gives exactly the smoothing penalty as mentioned above [4–7]. Alternatively, the second-order penalty

5 $$\begin{aligned} J_j(\beta _j) = \sum _{l=2}^{k_j-1} ((\beta _{j,l+1} - \beta _{jl}) - (\beta _{jl} - \beta _{j,l-1}))^2 = \sum _{l=2}^{k_j-1} (\beta _{j,l+1} - 2\beta _{jl} + \beta _{j,l-1})^2, \end{aligned}$$

can be used [14]. One of the benefits of considering ordinal predictors along with quadratic difference penalties in the framework of generalized additive models is that after implementing basis (3) in the appropriate way, gam() from mgcv can be used directly to fit a generalized linear/additive model with ordinal predictor(s) as needed for the BPD data. Besides pure model fitting, however, this provides us with additional tools; in particular, built-in estimation of the penalty/smoothing parameter(s) via (restricted) maximum likelihood ((RE)ML), further statistical inference, such as confidence intervals, and checking significance of smooth terms. Those tools utilize the mixed model and Bayesian interpretation of quadratic smoothing penalties on basis coefficients such as (4) and (5); compare [18, 22, 23] for details.

Add-on functions implementing the ordinal basis for use within mgcv have been made publicly available through R package ordPens [24]. After installing and loading ordPens, the gam() function from mgcv can be used with smooth terms s(..., bs = “ordinal”, m = 1) or s(..., bs = “ordinal”, m = 2) for the first- and second-order penalty, respectively. See the ordPens manual (R function ordSmooth()) for details and examples. To investigate whether the p-values of Wald-type tests with respect to smooth terms as provided by summary.gam() are reliable if using the ordinal basis/smoothing penalty, we used the confounder model, i.e., the model with information on bacteria removed, to estimate BPD probabilities. That means, the null hypothesis that the effect of (ordinal, bacteria-specific predictor) *x* is zero, is true by construction in this hypothetical model, because fitted BPD probabilities do not depend on *x*, given the other covariates. Using those probabilities, we simulated ‘new’ BPD response data, fit the model with smooth ordinal *x* added (and smoothing parameter estimated by REML), and stored the p-value of *x*. For *x*, we used information on gram negative/positive and pathogenic bacteria, respectively. As noted above, the corresponding ordinal factor gives the week colonization by the respective type of (oral) bacteria was detected for the first time. For each type of *x*, this was repeated 1000 times.

### Results

**Fig. 1:**
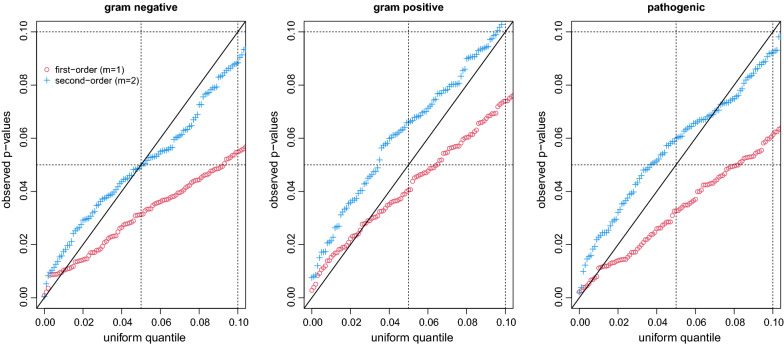
QQ-plots of p-values for frst- and second-order penalty (red/blue)

Figure 1 shows QQ-plots of the p-values observed on the simulated data employing the first- and second-order penalty, respectively. Since the distribution of smaller p-values is particularly relevant when testing with usual $\alpha \le 0.1$, we restrict plotting to that area. It is seen that p-values obtained when employing the first-order penalty (red) are typically too small. Problems with the first-order penalty can be explained by the fact that the null space of the corresponding smooth term has dimension zero (compare the mgcv manual). In other words, the null hypothesis in the framework of mixed models (which is used for estimation here), a zero variance component, is on the boundary of the parameter space, which means that standard theory does not apply [10, 11]. Results for the second-order penalty (blue), by contrast, look very encouraging. Consequently, we will only report results on the actually observed BPD data for the second-order penalty below. In earlier analyses [2], separate models were fit for each type of bacteria, and the first two weeks were collapsed to make (unpenalized) model fitting with dummy-coded, ordinal factors feasible. Thanks to the penalties presented here, we are now able to include all three predictors jointly while using all information with the resolution available.

**Table 1:** Results for parametric and smooth terms in the full and reduced model when using the second-order ordinal smoothing penalty

	Full model			
Parametric terms				
Covariate	Estimate	Std. error	z-value	p-value
(Intercept)	6.214	2.630	2.363	0.018
Weight (g)	− 0.013	0.004	− 3.381	<0.001
SGA sym.	1.909	1.359	1.405	0.160
Sex (male)	3.022	1.114	2.712	0.007
Multiples	1.524	0.744	2.048	0.041
Steroids	− 0.241	0.090	− 2.684	0.007
Antibiotics	0.079	0.090	0.874	0.382

Smooth terms				
Predictor	edf	Ref.df	Chi.sq	p-value
Gram negative	1.000	1.000	1.307	0.253
Gram positive	3.708	4.393	5.227	0.264
Pathogenic	5.258	5.831	13.711	0.030

	Reduced model			
Parametric terms				
Covariate	Estimate	Std. error	z-value	p-value
(Intercept)	6.426	2.170	2.961	0.003
Weight (g)	− 0.012	0.003	− 3.859	< 0.001
SGA sym.	1.991	1.290	1.544	0.123
Sex (male)	2.107	0.834	2.527	0.012
Multiples	1.054	0.528	1.995	0.046
Steroids	− 0.174	0.072	− 2.432	0.015
Antibiotics	0.079	0.078	1.013	0.311

Smooth terms				
Predictor	edf	Ref.df	Chi.sq	p-value
Gram negative	–	–	–	–
Gram positive	–	–	–	–
Pathogenic	4.973	5.696	13.573	0.027

**Fig. 2:**
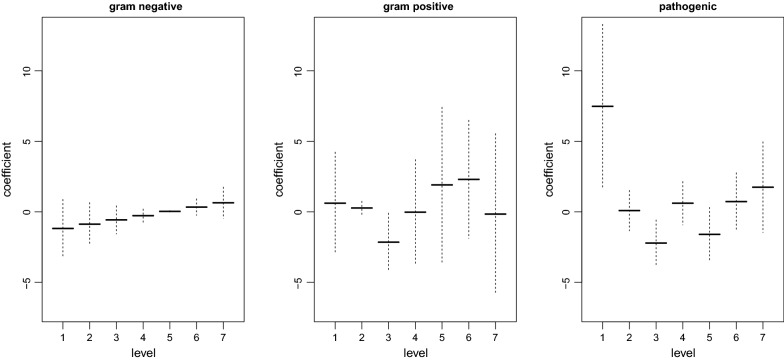
Fitted coefficients of ordinal predictors together with pointwise 95% confidence intervals in the full model with second-order penalties

Table 1 (top) shows the results for the parametric terms if using the second-order penalty (5) for the smooth terms. In particular, it is seen/confirmed that low birth weight is a risk factor for BPD, and also male infants and multiples have an increased risk of developing BPD. Antenatal steroids, by contrast, may decrease the risk. Results for the different types of bacterial colonization, which are included as ordinal predictors with smooth effects, are also given in Table 1 and Fig. 2. We see that the only significant effect is detected for pathogenic bacteria. The fitted function (Fig. 2, right) gives the impression that early detection is associated with increased risk of BPD. Statistical uncertainty, however, is very large (due to the small number of samples with week/level 1) as indicated by the confidence interval. When excluding information on gram negative and positive bacteria from the model, results for the remaining terms (Table 1, bottom) as well as fitted functions/coefficients for pathogenic bacteria (not shown) look very similar as before. In summary, our results using the ordinal smoothing approach are in line with earlier analyses [2], but allow for considering all three ordinal predictors (gram negative/positive, pathogenic bacteria) jointly, without the need to collapse levels.

## Limitations

With respect to the application/BPD data, the main limitation is the small number of samples in week 1 for pathogenic bacteria. Since the shape of the function in Fig. 2 (right) depends on the coefficient for week/level 1, this shape should not be over-interpreted here. From a technical point of view, if using the second-order penalty (5), a problem can occur with confidence intervals in terms of under-coverage if the fitted coefficient function is close to being linear (compare Fig. 2, left). This problem is also found for (generalized) additive models with continuous covariates, and the suggested fix is to change the target of inference to the smooth term plus the overall model intercept [18, 25]. Furthermore, our implementation does not include extensions like ordinal smoothing spline *isotonic* regression [7]. Finally, it should be noted that all statements made and conclusions drawn in this article refer to statistical inference if smoothing parameters are estimated by REML (or ML). When using GCV (which is the default in mgcv!), results should be treated with caution.

## Data Availability

The software presented together with examples is publicly available on CRAN through open source R add-on package ordPens [24]. The data [2] that support the findings of this study are available on reasonable request from H.E. (harald.ehrhardt@paediat.med.uni-giessen.de). An earlier yet extended version of this article providing further technical details and simulation studies is available at https://arxiv.org/abs/2102.01946.

## Abbreviations

BPD: Bronchopulmonary Dysplasia
(RE)ML: (Restricted) maximum likelihood
GCV: Generalized cross-validation
